# Sleep Quality in Rheumatoid Arthritis: Relationship Between the Disease Severity, Depression, Functional Status and the Quality of Life

**DOI:** 10.4021/jocmr1648w

**Published:** 2013-12-13

**Authors:** Mustafa Akif Sariyildiz, Ibrahim Batmaz, Mehtap Bozkurt, Yasin Bez, Mehmet Guli Cetincakmak, Levent Yazmalar, Demet Ucar, Tahsin Celepkolu

**Affiliations:** a Department of Physical Medicine and Rehabilitation, Dicle University Faculty of Medicine, Diyarbakir, Turkey; b Department of Psychiatry, Dicle University Faculty of Medicine, Diyarbakir, Turkey; c Department of Radiology, Dicle University Faculty of Medicine, Diyarbakir, Turkey; d Department of Family Physician, Dicle University Faculty of Medicine, Diyarbakir, Turkey

**Keywords:** Rheumatoid arthritis, Sleep quality, Disease activity, Depression

## Abstract

**Background:**

The aim of this study was to evaluate sleep quality and the related variables in patients with rheumatoid arthritis (RA).

**Methods:**

Ninety-four patients diagnosed with RA and fifty two healthy controls were enrolled in the study. Disease activity was assessed through the Disease Activity Score (DAS) 28 scale. All patients were assessed using the Rheumatoid Arthritis Quality of Life and Health Assessment Questionnaire scales, together with the Beck Depression Inventory. Radiological damage was calculated with the modified Larsen method. The Pittsburgh Sleep Quality Index (PSQI) was used for the evaluation of the sleep disturbance.

**Results:**

The patients with RA had significantly higher scores in the subjective sleep quality, sleep latency, habitual sleep efficiency, sleep disturbance domains and the total PSQI score compared to the healthy control group. According to the results of Spearman’s analysis, there was a significantly correlation between the age, disease activity, CRP, pain, fatigue, depression, functional disability, quality of life, radiological damage, menopause status, duration of morning stiffness, ESR levels and the sleep disturbance. The logistic regression analysis indicated that depression and DAS 28 scores were predictors for poor sleep quality.

**Conclusion:**

The sleep quality is disturbed in patients with RA. The poor sleep quality is especially associated with the disease activity and depression.

## Introduction

Rheumatoid arthritis (RA) is a chronic autoimmune disorder characterized by joint pain, joint swelling, morning stiffness, fatigue, depression, sleep disturbances and disability [1-4]. Several studies that have pointed out sleep disturbances in RA patients have revealed that 54% to 70% of RA patients report problems related to their sleep, including difficulty falling asleep, poor sleep quality, non restorative sleep, wakefulness, awakenings during the night and excessive daytime sleepiness [5-8]. Conversely, sleep disturbance may contribute to the pain, disease activity, stiffness, fatigue and mood disturbances in affected patients [7, 9-11].

Possible causes of sleep disturbance in RA include inflammatory disease activity, joint pain, sleep apnea, restless leg syndrome and psychological disorders like depression [2, 12]. Wolfe et al [12] have revealed that pain and depression are independently associated with sleep disturbances in RA. Nicassio and Wallston [13] have shown that sleep disturbance is associated with depression independently of pain, and that long-term pain is a predictor of deteriorating sleep disorders.

Sleep quality is an important component of the quality of life, and it is a known fact that sleep disturbances affect the quality of life in patients with RA [14]. It has also been demonstrated that the higher depression, fatigue and pain scores in patients with RA are related to functional disability [15, 16] Wolfe et al [16] have revealed that pain and depression are important determinants of the functional disability in patients with RA. Poor sleep quality may contribute to the feelings of pain, fatigue and mood disturbances and may further deteriorate the functional ability and the quality of life.

The aim of this study is to determine the influence of RA on sleep quality and to reveal the relationship of the laboratory parameters, radiological damage, disease activity, functional status, quality of life and the psychological state with the sleep disturbance.

## Material and Method

### Participants

Ninety-four patients who applied to the Physical Medicine and Rehabilitation outpatient clinic in university hospital in Turkey between October 2011 and May 2012 and diagnosed with RA according to the 1987 American College of Rheumatology [17] criteria were included in this study. The patients were between the ages of 18 and 65. All the patients were married and have regularly been using DMARD and/or anti-TNF for the last three months. Fifty two age- and sex-matched healthy subjects were also enrolled as the control group. The present study had a cross-sectional design approved by the local ethics committee. All the recruited subjects signed an informed consent form before participating in the study.

Patients with thyroid disorders, infections, those who had undergone a hysterectomy or who had any chronic diseases such as diabetes mellitus, heart or renal failure, as well as those with a history of serious psychiatric disorders were excluded from study.

### Measurement of the clinical variables

The demographic and clinical characteristics of the patients such as age, sex, height, weight, educational level, the disease duration (which is defined as the duration since the onset of the first symptoms of RA), degree of morning stiffness, tender-swollen joint count and the physician’s and patient’s global assessments were determined. Drug use (Non steroidal anti-inflammatory drugs (NSAIDs), disease-modifying anti-rheumatic drugs (DMARDs), anti-tumour necrosis factor (TNF), or corticosteroids) and the smoking status were recorded. The duration of the morning stiffness (minutes), mean pain and mean daytime fatigue (100 mm visual analogue scale (VAS)) were also noted. The disease activity (DAS28) scores were evaluated using the disease activity score calculator [18]. The erythrocyte sedimentation rate (ESR) was measured through the Westergren method (mm/h) and the serum C-reactive protein (CRP) level was determined with the help of nephelometry (mg/dL). The RF titres were also measured through the nephelometric immunoassay method (IU/mL).

### Functional disability, radiological score and the quality of life

The quality of life was evaluated using the disease-specific rheumatoid arthritis quality of life (RAQoL) scale [19]. The health assessment questionnaire (HAQ) was used for the evaluation of the functional status [20]. Standardized radiographs of both the hands and feet were performed for all the RA patients and the degree of joint damage were assessed by a radiologist according to Larsen’s method [21].

### Measurements of the psychological variables

The Beck depression inventory (BDI) was used for evaluation of the depression in patients with RA. BDI is a likert-type, self-reported questionnaire which evaluates the level of depression according to the replies to 21 questions [22]. In our study, a BDI score of 13 was chosen as the cut-off point for depression as recommended [23]. The validity and reliability of the scale’s Turkish version have already been shown as satisfactory [24]. The results were evaluated by a psychiatrist.

### Measurement of sleep quality

The Pittsburgh Sleep Quality Index (PSQI) measures the patient’s self-reported sleep quality over the last month [25]. The scale has 19 items and measures 7 components of sleep quality: subjective sleep quality, sleep latency, sleep duration, habitual sleep efficiency, sleep disturbances, use of sleeping medication, and daytime dysfunction. A global PSQI score corresponding to the total of the individual scores from the 7 components is calculated (range = 0 - 21). A result equal to 6 or above is considered indicative of poor sleep quality. The test has high diagnostic specificity for detecting clinical sleep impairment. The Turkish version of this scale has been verified in terms of its validity and reliability by Agargun et al [26].

### Statistical analysis

The calculations were performed using the Statistical Package for Social Sciences for Windows software version 16.0. The Kolmogorov-Smirnov test was used to confirm that data within the ranges of normal distribution in both groups. A non-parametric test was employed for the variables outside the normal distribution. The comparison of the data between reciprocal groups was carried out through the Mann Whitney-U test. The independent-sample t-test was used to examine the differences in sleep quality, pain, depression, fatigue, and functional disability between those taking certain RA medications and those who do not. Correlations between sleep disturbance, depression status and the RA-related variables were investigated with the help of Spearman’s correlation. By using a binary logistic regression analysis, a multivariate analysis was performed to detect independent predictors for the occurrence of sleep disturbances and to find out confounding effects between potentially independent predictors. A forward stepwise method was used to construct multivariate logistic regression models in relation to various dependent variables. Statistical significance was based on a value of P < 0.05 with a 95% confidence interval.

## Results

The clinical and demographic characteristics of the patients and the healthy controls are listed in Table 1. The majority of the RA patients were female (81.5%). The mean age was 46.20 ± 12.32 years. According to the suggested cut off point for the PSQI global score (scores > 5), sleep disturbances were observed in 64.1% of the patients with RA. Among the patients, 43.3% had depression symptoms (BDI > 13) and 48% were postmenopausal. The patients with RA had significantly higher scores in the subjective sleep quality, sleep latency, habitual sleep efficiency, sleep disturbance domains and in terms of the total PSQI score compared to the healthy control group (P < 0.05) (Table 2).

**Table 1:** Clinical and Demographic Characteristics of the Patients With RA and Healthy Controls (Mean ± SD)

Disease characteristics	RA patients (n:94)	Controls (n:52)	P
Age (years)	46.20 ± 12.32	44.07 ± 9.75	NS
Female	81.5%	79.2%	NS
Postmenopausal patient	48%	45%	NS
Disease duration (years)	4.66 ± 5.14		
Duration of morning stiffness (minute)	62.99 ± 57.78		
Pain (VAS, mm)	5.14 ± 2.65		
Fatigue (VAS, mm)	4.68 ± 2.56		
ESR (mm/h)	29.18 ± 21.71		
CRP (mg/dL)	3.28 ± 6.26		
RF (IU/mL)	120.57 ± 176.25		
HAQ	1.08 ± 0.87		
DAS28	4.17 ± 1.59		
Depression (BDI score)	15.56 ± 8.75 (53.3%)		
RAQoL	14.05 ± 10.19		
Larsen score	30.47 ± 19.70		
NSAID	62%		
Anti-TNF	48%		
DMARD	72%		
Corticosteroid	42%		

VAS: visual analog scale; ESR: Erythrocyte sedimentation rate; CRP: C-reactive protein; RF: rheumatoid factor; HAQ: health assesment questionnaire; DAS: disease activity score; BDI: Beck depression inventory; RAQoL: rheumatoid arthritis quality of life; RA: rheumatoid arthritis; n: number of cases; SD: standard deviation.

**Table 2:** Mean Scores of the RA Patients and Controls for the PSQI Domains (Mean ± SD)

	RA Patients (n:94)	Controls (n:52)	P
Subjective sleep quality	1.61 ± 0.86	0.81 ± 0.56	< 0.001
Sleep latency	1.36 ± 0.96	0.77 ± 0.73	< 0.001
Sleep duration	1.14 ± 0.87	1.00 ± 0.65	0.274
Habitual sleep efficiency	0.73 ± 0.99	0.21 ± 0.63	< 0.001
Sleep disturbances	1.24 ± 0.81	1.00 ± 0.56	0.04
Use of sleeping medication	0.10 ± 0.38	0.11 ± 0.42	0.925
Daytime dysfunction	0.80 ± 0.78	0.73 ± 0.69	0.56
PSQI total	7.03 ± 3.97	4.60 ± 2.64	< 0.001

SD: standard deviation; PSQI: Pittsburgh sleep quality index; RA: rheumatoid arthritis; n: number of cases.

According to Spearman’s analysis, there was a significantly higher correlation between the age, pain, fatigue, DAS28, CRP, BDI, HAQ, RAQoL, the Larsen scores and the sleep disturbance (P < 0.01). There was also a significantly lower correlation between the menopause status, duration of morning stiffness, ESR levels and the sleep disturbance (P < 0.05). Correlations between the disease-related variables, individual domains and the total PSQI scores are listed in Table 3.

**Table 3:** Correlation Between the PSQI Scores With Clinical, Radiological, Laboratory and the Psychological Characteristics in Patients With RA

Characteristics	Subjective sleep quality	Sleep latency	Sleep duration	Habitual sleep efficiency	Sleep disturbances	Use of sleeping medication	Daytime dysfunction	PSQI total
Age	r: 0.110	r: 0.325**	r: 0.255*	r: 0.205	r: 0.382**	r: -0.035	r: 0.311**	r: 0.308**
Disease duration	r: -0.060	r: 0.119	r: 0.012	r: 0.149	r: 0.053	r: 0.138	r: 0.099	r : 0.103
Postmenopausal patients (versus premenopausal)	r: 0.079	r: 0.306**	r: 0.121	r: 0.204	r: 0.381**	r: 0.014	r: 0.162	r: 0.251*
Duration of morning stiffness	r: 0.229*	r: 0.250*	r: 0.139	r: 0.166	r: 0.298**	r: 0.172	r: 0.093	r: 0.218*
Pain ^VAS^	r: 0.256*	r: 0.385**	r: 0.305**	r: 0.285**	r: 0.335**	r: 0.072	r: 0.115	r: 0.371**
Fatigue ^VAS^	r: 0.249*	r: 0.235*	r: 0.209*	r: 0.351**	r: 0.324**	r: 0.-002	r: 0.319**	r: 0.354**
ESR (mm/h)	r: 0.235*	r: 0.300**	r: 0.124	r: 0.177	r: 0.275**	r: -0.060	r: 0.181	r: 0.263*
CRP (mg/dL)	r: 0.276**	r: 0.256*	r: 0.191	r: 0.189	r: 0.344**	r: 0.000	r: 0.155	r: 0.302**
RF	r: -0.193	r: -0.088	r: -0.043	r: 0.015	r: -0.012	r: 0.004	r: -0.190	r: -0.154
HAQ	r: 0.368**	r: 0.360**	r: 0.301**	r: 0.243**	r: 0.397**	r: 0.063	r: 0.322**	r: 0.431**
DAS28 (Disease activity score)	r: 0.358**	r: 0.369**	r: 0.285**	r: 0.289**	r: 0.405**	r: 0.018	r: 0.113	r: 0.375**
BDI	r: 0.385**	r: 0.361**	r: 0.406**	r: 0.316**	r: 0.373**	r: 0.171	r: 0.299**	r: 0.489**
RAQoL	r: 0.305**	r: 0.331**	r: 0.321**	r: 0.324**	r: 0.379**	r: 0.106	r: 0.283**	r: 0.424**
Larsen score	r: 0.095	r: 0.249*	r: 0.260*	r: 0.323**	r: 0.176	r: 0.090	r: 0.121	r: 0.292**

VAS: visual analog scale; ESR: Erythrocyte sedimentation rate; CRP: C-reactive protein; RF: rheumatoid factor; HAQ: health assesment questionnaire; DAS: disease activity score; BDI: Beck depression inventory; RAQoL: rheumatoid arthritis quality of life; PSQI: Pittsburgh sleep quality index; RA: rheumatoid arthritis. *P < 0.05, **P < 0.01.

When the patients were compared in terms of the medication status (anti-TNF, DMARD, corticosteroids, or none), no significant difference has been observed between the treatment modality and the PSQI scores (P > 0.05). The logistic regression analyses indicated that BDI and DAS 28 scores were predictors for poor sleep quality (P < 0.05) (Table 4).

**Table 4:** Final Logistic Regression Model; PSQI Total Score as Dependent Variable (PSQI ≥ 6 Versus PSQI < 6)

	B	SE	Wald	P	OR	95% CI for OR	
						Lower	Upper
BDI	-0.124	0.044	8.071	0.004	1.132	1.039	1.234
DAS28	-0.706	0.207	11.65	0.001	2.024	1.351	3.039
Constant	3.957	0.965	16.798	0.000	0.019		

DAS: disease activity score; BDI: Beck depression inventory; B: coefficients; SE: standard error; OR: odds ratio; CI: confidence interval.

## Discussion

In this study, the mean PSQI scores indicate poorer sleep quality in the patients with RA compared to the controls. The logistic regression analyses have pointed out the disease activity and depression as the predictors of poor sleep quality. In various studies conducted in patients with RA, depression has been reported to have a negative influence on the quality of sleep and it has also been described as a risk factor for sleep quality [9, 12, 13, 27]. The pain in the patients with RA leads to sleep disturbances, while the constant pain and sleep disturbances increase the patient’s propensity to depression [13, 28]. The multivariate regression analyses performed in a longitudinal study by Nicasso et al [13] have demonstrated that severe pain and severe sleep disorders are independently associated with depression in the patients with RA. These results are also in line with our study and point out that the depression contributes to sleep disorders in patients with RA.

In previous studies, pain at rest and joint stiffness were determined to be factors associated with sleep disturbances [5, 13, 15, 29]. It has also been reported that the duration of morning stiffness affects the quality of sleep [7, 15, 30, 31]. In a recent study, Luyster et al [3] found that poorer sleep quality is associated with greater pain severity. Aside from these findings, it has also been shown that the sleep quality is also positively influenced when the active inflammatory disease and the resulting pain are treated and the arthritic process is stopped [32, 33]. Many investigators have observed that any increase in the disease activity is related with sleep disorders in patients with RA [7, 12, 34, 35]. There is also a study where the disease activity was found to be unrelated with sleep disorders, but this conflicting result may be due to the small number of patients that participated in the study (n = 19) [8]. The DAS 28 score used to measure the disease activity in the patients with RA is related to the number of the tender and swollen joints. A greater number of tender and swollen joints may cause frequent interruptions during the sleep throughout the night and lead to longer durations of morning stiffness (in our study, the mean duration of morning stiffness was 62 minutes). Consequently, since getting up and moving around may get problematic, the sleep quality is negatively affected. Also in our study, the disease activity, greater pain, and morning stiffness were found to be associated with sleep disorders.

Mahowlad et al [36] explored sleep in RA patients using polysomnography and suggested that fatigue in RA may be a manifestation of sleep fragmentation. Similarly, sleep quality has also been recognized as a predictor of fatigue in older adults with RA [15]. In a recent study, Fuyster et al [3] revealed that poor sleep quality is associated with higher levels of fatigue. In patients with RA, while the fatigue may disturb sleep quality, the disturbance in the sleep quality may also lead to greater fatigue [37]. In accordance with these studies, also in the present study, higher fatigue levels were observed to be associated with sleep disturbance according to the PSQI index.

In this study, a higher Larsen score was also correlated with poor sleep quality. The association of the radiological damage with the sleep quality were not investigated in the previous studies. On the other hand, there are various studies focussing on the relationship between the radiological damage, and the disability and quality of life in the patients with RA. Some studies have pointed out that the influence of the radiological damage on the quality of life was less severe compared to pain, depression, disease activity and the functional status [38, 39]. At the same time, Prajs et al [40, 41] have suggested that radiological damage and functional status affect the quality of life in RA more than the disease duration and disease activity. In accordance with these results, our study revealed the influence of the radiological damage on poor sleep quality in RA patients. Since radiological damage is associated with the disease activity and the low quality of life, it may contribute to sleep disturbances.

Higher RAQoL and higher HAQ scores were also correlated with sleep disturbances in the present study. Many patients with RA experience difficulties in the activities related to daily life. This causes greater fatigue during the day, which leads to interrupted sleep at night, thus to sleep disturbances. Also, the inability to perform the daily activities may cause depression and lead to sleep disturbance in patients with RA [42]. In RA populations, fatigue, pain and depression have been found to be associated with the physical disability [15, 42]. Wolfe et al [16] have assessed functional disability using the Health Assessment Questionnaire (HAQ) and found pain and depression to be important determinants of functional disability in 1843 patients with RA. Based on the links between sleep, pain, depression and disability; an indirect pathway may exist between the sleep quality and functional disability in RA [3]. The causal relationships between pain, fatigue, depression and disability may be reciprocal. While the pain, fatigue and depression may interfere with the activities related to daily life and thus cause sleep disorders and disability, sleep disorders may also contribute to the pain, fatigue and depression and lead to disability [3].

In our study, Spearman’s analyses revealed that the postmenopausal status of the patients was correlated with the sleep disturbances. Also, 78% of the postmenopausal RA patients described hot flashes. Sleep quality may also be disturbed in postmenopausal women. Approximately 70% of healthy women experience hot flashes. Hot flashes occur as a result of the increase in the plasma epinephrine and brain norepinephrine levels besides other autonomic changes such as increased heart rate, palpitations, and feelings of anxiety or panic [43, 44]. Healthy women who experience hot flashes have more frequent sleep disturbances [45].

The relationship between the medication and sleep disorders in RA is unclear. Placebo controlled studies have demonstrated that NSAIDs increase sleep quality [46, 47]. However, polysomnographic results have indicated that the administration of NSAIDs does not change the sleep architecture, sleep latency or sleep efficiency in patients with RA [48]. However, there are also studies showing that anti-TNF agents increase the sleep quality in patients with RA [49, 50]. In their prospective and controlled study, Wells et al [49] have reported greater improvement in patients using abatecept in comparison to the patients using methotrexate. In the present study, no relationship was detected between the RA medication and sleep quality.

We have observed the sleep disorder to be positively correlated with the patients’ age in our study. The relationship of the age with the sleep quality in the patients with RA is yet to be cleared. Bixler et al [51] have reported that the age is correlated with shorter sleep durations. In another study, the sleep quality in patients with OA and RA was compared and the sleep disorders in patients with OA were attributed to the age [27]. Still, some studies have failed to reveal any relationship between the age and the sleep disorder [3, 9].

The limitation of this study was its cross-sectional design. Prospective studies are needed in order to reveal the relationship between disease activity, depression, pain, functional disability and sleep quality. Besides, we have evaluated the sleep quality through a questionnaire, while a polysomnographic evaluation could have supplied more accurate results.

### Conclusion

As far as we know, this is the first study evaluating correlation of the radiological scores and the sleep quality in patients with RA. Also, the effect of the disease activity and depression on the sleep quality has been demonstrated by means of the logistic regression model in this study. Poor sleep quality was observed in RA patients. Sleep disturbances in patients with RA occur due to multifactorial origins and multidirectional disease-related variables like the disease activity, depression, pain, fatigue, functional disability, radiographic score, quality of life and the CRP levels.
